# Bringing population mobility into focus to achieve HIV prevention goals

**DOI:** 10.1002/jia2.25136

**Published:** 2018-07-19

**Authors:** Carol S Camlin, Susan Cassels, Janet Seeley

**Affiliations:** ^1^ Department of Obstetrics, Gynecology and Reproductive Sciences Department of Medicine University of California San Francisco USA; ^2^ Department of Geography University of California Santa Barbara USA; ^3^ Department of Global Health and Development London School of Hygiene and Tropical Medicine London UK

**Keywords:** Population mobility, Migration, Universal testing and treatment, Structural drivers, Prevention, Key and vulnerable populations

The ambitious UNAIDS ‘90‐90‐90' targets, aiming to end the AIDS epidemic by the year 2030 1, are a response to the profound therapeutic and preventive benefits of HIV treatment. Universal voluntary HIV counselling and testing followed by prompt initiation of antiretroviral therapy (ART) for all those diagnosed HIV‐infected, an approach known as Universal Test and Treat (UTT), is now seen as the primary means through which the 90‐90‐90 targets can be achieved. Beyond the targets, the recently emergent concept of a prevention cascade 2 also recognizes the imperative of “coverage” for achieving population‐level effects of HIV prevention interventions, which are needed together with the expansion of treatment to end the epidemic 3.

Perhaps nowhere else in the world is more momentum required to bring HIV treatment *and* prevention to all who need it than in Africa. The HIV epidemic on the continent has been concentrated in eastern and southern Africa, where an estimated 19.4 million adults are living with HIV 4. Despite substantial progress in recent years, only approximately 60% of people living with HIV (PLHIV) in the region were receiving ART in 2016 4; an estimated 45% had a suppressed viral load as a result of their successful engagement in care in 2015 5.

These estimates reflect tremendous successes of HIV prevention and care initiatives towards accelerating ART coverage for sub‐Saharan African populations, and many successful efforts to reach key populations—yet also the sobering reality that what has been achieved to date has been achieved in easier to reach populations. A focus by UNAIDS and global leadership on harder to reach, key populations, that are particularly important for further scale‐up, is laudable; but to date, inadequate attention has been paid to population mobility, a major force that challenges the HIV care cascade and threatens the promise of “treatment as prevention” particularly in high HIV prevalence, resource‐limited regions. Ultimately, strategies to attain the 90‐90‐90 targets that do not account for the complex dynamics of mobility in specific settings will fail to engage successfully with the magnitude of populations necessary to end the epidemic 6.

There is an urgent need to deepen our understanding of population mobility, its heterogeneous forms and gender dimensions in high HIV prevalence areas, its effects on sexual behaviour and sexual networks, HIV testing and engagement in HIV care and treatment, and on HIV acquisition and onward transmission, even as widespread HIV prevention interventions are underway—indeed, *especially* in contexts in which interventions such as UTT are underway. To understand whether and how mobility attenuates the effectiveness of HIV prevention and care initiatives is a vital first step towards identifying the full set of solutions, inclusive of mobile populations, that will be needed to end the epidemic.

Historically, the spread of HIV in sub‐Saharan Africa followed the corridors of population movement, as people moved to expanding urban areas and other places of employment opportunity 7, 8, 9, 10. Today, mobility continues to place individuals at great risk of HIV acquisition as well as onward transmission 11, 12, 13. The highly gendered nature of that linkage is well‐documented, yet rarely commented upon. Forms of mobility in sub‐Saharan Africa are diverse and complex relative to those of other regions, and men's and women's patterns of movement significantly differ, with women increasingly participating in mobility 14, 15, 16, 17, 18, as has been seen elsewhere 19, 20, 21, 22. While transcontinental migration from Africa is low 23, the intra‐sub‐Saharan African emigration rate represents the largest south to south movement of people in the world 24. Data for the study of internal migration have been limited; however, data from Demographic Surveillance Sites show that 7% to 20% of local populations in surveillance areas, often over 30% of young adults, have migrated annually in recent years 14, 25. Rural to urban migration flows do not predominate in all settings 26; rather, counter‐urbanization 27 and circulation between rural areas, semi‐urban towns, and rural perimeters of cities are common 14. Mobility has positive benefits for development and is a key driver of economic growth in southern and eastern Africa: from 50% to 80% of rural households have at least one migrant member 24, and the “sending” of a female migrant has been particularly advantageous to the poorest households 23, 28, 29.

Despite its importance, research on the effects of mobility in sub‐Saharan Africa on HIV care engagement, and on effectiveness of ART as treatment and as prevention, is in its nascency. Existing literature mostly from the global north has shown that mobility can break bonds between individuals and HIV care systems 30, thus leading to disengagement from care, treatment interruption and poor health outcomes. Research on mobility and care engagement in sub‐Saharan African settings is relatively scarce; thus there are key questions about the extent to which mobility is contributing to the gap that exists between the remarkable promise of ART for large‐scale prevention 31, 32, 33, and actual engagement in care 34.

Recent meta‐analyses in sub‐Saharan African populations have shown that 30% to 60% of people living with HIV are lost to follow‐up at each step after HIV diagnosis 35, 36, 37. People repeatedly exit and re‐enter the care cascade at various points 38, 39. Greater attention to the contextual factors that shape HIV treatment experiences, engagement and outcomes has recently emerged in social science research on HIV 40, revealing the complex social realities underlying “bottlenecks” in the cascade 40. Yet data have been limited for investigating the role of mobility in these care cascade shortfalls.

Several meta‐analyses and systematic reviews of the literature examining factors associated with care entry, engagement and retention 35, 37, 41, 42, 43 show that optimal lifelong engagement in HIV care can be threatened by a range of factors at the individual, social, and structural levels. Mobility affects many of the factors found to contribute to delayed entry or lapses in care, including psychological factors (e.g. seeking care away from home because of stigma) 39, 44, 45, clinic characteristics (e.g. waiting times) 46, 47 and structural barriers 44, 48 such as distance to clinic and transportation costs 44, 47, 49, 50. Yet the direct impact of mobility, and the pathways for this impact, have not been examined in depth 30, 51. Better measures and methods are needed to understand how mobility affects the ability of individuals to successfully navigate the HIV care cascade. This is especially urgent for sub‐Saharan Africa, where levels of mobility are high and forms of mobility are complex and dynamic. The effects of mobility on initiatives designed to extend the reach of primary HIV prevention interventions in these settings are, to date, unknown— a glaring gap in the literature.

The articles in this supplement of the *Journal of the International AIDS Society* focus on crucial questions regarding the forms and dimensions of mobility and the impact on HIV prevention and care initiatives—particularly in Southern and Eastern Africa, but also in the ‘receiving’ nations in the global north of sub‐Saharan African migrants. This volume includes contributions from each of the large‐scale UTT studies in five African countries 52, which include HPTN 071 Population Effects of Antiretroviral Therapy to Reduce HIV Transmission (PopART), Sustainable East Africa Research in Community Health (SEARCH); the MaxART (Early Access to ART for All) implementation study; and the ANRS 12249 Antiretroviral Treatment as Prevention (TasP) trial. It also includes contributions from studies outside UTT trial settings on the impacts of mobility on care engagement. The authors of the papers use different methods and approaches to identify emergent themes, key problems and gaps in the study of mobility and HIV prevention and care and point to potential solutions. The collection includes quantitative research that advances approaches towards measurement of diverse forms of mobility and its impacts, and also highlights the value of qualitative research for understanding the meaning of factors driving linkage to HIV treatment and prevention in the context of migration and mobility.

We observe four major themes across the contributions to this volume: first, the spatial *and* temporal dimensions of mobility are important, because mobility is inherently linked to time exposed to interventions and services within geographies; secondly, types of mobility in different populations and settings are diverse and highly gendered, and this heterogeneity across contexts should inform HIV prevention and treatment approaches in specific settings; thirdly, while mobility is intertwined with other factors that affect engagement in HIV care and prevention, it often sets into motion a “chain of events” that leads to disengagement; and fourth, the period following resettlement in new destinations is one of instability, in which risk is heightened, disrupting engagement and leading to ‘missed opportunities’ for progress along the prevention and care cascades. Such missed opportunities must be addressed through health systems‐ and policy‐level actions. In sum, this collection speaks to a need for reconfiguration of programs and services to respond to the challenges to HIV care and prevention engagement that are presented by the mobility of populations.

Speaking to the dynamic nature of mobility and the importance of temporality, Larmarange and colleagues 53 present findings from the ANRS 12249 Antiretroviral Treatment as Prevention (TasP) trial, that highlight the effect of population mobility on temporality of and geographic exposure to the UTT intervention in rural KwaZulu‐Natal, South Africa. The TasP cluster‐randomized trial failed to show a reduction in HIV incidence at population level when ART was proposed regardless of CD4 count. The authors describe a dynamic cascade of various degrees of exposure to the trial interventions, using both calendar (population) and exposure (individual) time approaches in their analyses, and show that the structural effects of mobility diluted the impact of the UTT strategy. In a context of high HIV incidence, the circulation of newly infected individuals in and out of communities slowed down TasP efforts to increase ART coverage and population viral suppression, ultimately attenuating any population‐level impact on HIV incidence.

In their study of population mobility embedded within the SEARCH trial in rural populations in Kenya and Uganda, Camlin and colleagues 54 describe significant heterogeneity in mobility across regions in Kenya and Uganda, which correlated with heterogeneous levels of risk behaviour and HIV prevalence observed across the regions: communities with higher proportions of mobile residents tended to also have higher HIV prevalence. Mobility may undermine UTT interventions in high prevalence areas if mobile populations are spending time exposed outside of their communities and indeed they were as follows: both migration and also localized short‐term mobility were associated with higher risk sexual behaviour, especially among women. Livelihoods requiring mobility for women, such as market trading, and the fish trade in particular, were strongly associated with their higher risk sexual behaviour. In contrast, men's labour‐related mobility was not associated with higher risk behaviour; their travel for other purposes (e.g. attending funerals, seeking care, visiting family), was. This work highlights the need for gender‐specific interventions among mobile populations.

The links between mobility and livelihoods that affect engagement in HIV care and prevention services and interventions also figure prominently in two contributions from the PopART trial. Bond and colleagues 55 used longitudinal qualitative data focused on the lives of six people living with HIV in urban Zambia to show the challenges of juggling household responsibility, livelihood mobility and HIV management. For five of the six, ongoing engagement in treatment could not be sustained because of travel. The authors highlight the need for differential care options which can adjust more to clients’ temporal and spatial realities. Hoddinott and colleagues 56 used ethnographic and participatory research to describe patterns of ‘household fluidity’ in the Western Cape of South Africa, and explored how movements in and out of households to ensure livelihoods and social support shape HIV service access. With their conceptualization of ‘fluidity’, the authors challenge conventional sociological concepts of households, that is, they are not static, contained or bounded as the typical measures would suggest. Echoing the recommendations of Bond and colleagues, the authors call for “responsive, flexible health service delivery systems designed to support continuity in care across many shifts in client circumstances.”

Shabalala and colleagues 57 present findings from a mixed methods study to improve understanding of care retention among ART clients in the MaxART implementation study in Swaziland. The authors examined associations between socio‐demographic characteristics and retention, and conducted in‐depth interviews with clients who were lost‐to‐follow‐up from the study to explore their reasons for leaving care. Mobility, particularly relocations to another community far from the facility where the client initially obtained ART, was described by study participants as the first step in a complex chain of events that affected retention in care. Several often‐intersecting reasons for discontinuing ART also mattered, including “harsh treatment by health care workers”, ART side effects, stigma, and food insecurity. These findings provide further evidence in support of Ware's conceptualization of care disengagement as a process through which missed visits, and ensuing reluctance to return, can erode patients' feelings of connectedness to care over time 39. They also expand upon this conceptualization by documenting how migration events can trigger the chain of events that leads to disengagement.

Expanding upon this theme, studies conducted outside of UTT trial contexts highlight how the period following resettlement in new destinations is a period of instability in which behavioural risks of HIV acquisition are heightened, and engagement in care and prevention is disrupted, leading to missed opportunities for improving the prevention and care cascades. Fakoya and colleagues 58 present findings from a study of care engagement among migrants living with HIV in Europe. Using data from 57 HIV clinics in nine countries, the authors investigated the barriers and facilitators to HIV testing, and assessed the current treatment and healthcare needs of migrants living with HIV in Europe. Noting that immigrants are overrepresented in the European HIV epidemic, the authors present evidence suggesting that exposure to HIV after migrating accounts for a substantial proportion of infections among migrants, and that opportunities for HIV prevention are being missed. They call for a greater attention to the HIV prevention needs of immigrants to Europe, including interventions to reduce HIV acquisition among migrant gay and bisexual men, and to expand HIV testing opportunities for migrant heterosexual men and women.

Two articles in this volume examine women's migration in the context of pregnancy. Clouse and colleagues 59 present findings from a mixed methods study to identify drivers and types of mobility among pregnant and postpartum women living with HIV in the Johannesburg area in South Africa. They examine long‐distance travel of mothers and infants before and after delivery, finding that the frequent mobility in the peripartum period “underscores the challenge of ensuring a continuity of HIV care in a fragmented health care system that is not adapted for a mobile population.” In another setting in South Africa (Cape Town), Phillips and colleagues 60 also examined the mobility of women attending an integrated antenatal‐ART clinic. Using routine electronic health data (including laboratory testing, ART dispensing and clinic visits) to measure the movements and access points of pregnancy and postpartum women, the authors show that a substantial proportion of women do not link to postpartum care, and among those who do, long‐term retention remains a challenge as women move to a wide variety of facilities locally and nationally.

Vearey 61 offers a Commentary that speaks to a need for improving the policy environment to address better the HIV prevention and care needs of mobile populations. She explores challenges and strategic opportunities for “re‐setting” the policy agenda on migration and HIV in southern Africa, specifically in the policy environment of the Southern African Development Community (SADC) – a region associated with high levels of migration, and home to the largest population of people living with HIV globally. Drawing upon policy review, empirical data, and on‐going participant observation within local, regional and global policy processes, Vearey shows how current policy processes have the potential to undermine efforts to improve the global responses to migration and HIV. She argues that, “without mainstreaming migration, HIV programmes will continue to struggle, and key health targets will not be met.”

Lastly, in our Viewpoint, the editors have expanded on two specific organizing concepts that have emerged from our work on this volume: the time scales of mobility, and migrants' sexual networks. Temporal scales of population mobility are complex, sexual risk behaviour can change in relation to the timing of migration, and timing of migration can interact with timing of, and exposure to, HIV prevention efforts. Additionally, the timing, sequence and spatial scale of migrant's sexual networks can mitigate intervention effectiveness. Cassels, Camlin and Seeley 62 argue that focusing on temporal patterns of mobility and network characteristics will not only help to explain why population mobility presents a challenge for HIV prevention and care, but can be leveraged to improve future interventions.

The collection of articles in this volume contribute to framing a future policy and research agenda. Treatment access is a key area of concern: how can migrants sustain their care in the myriad destinations to which they travel? What interventions, policies, or health systems improvements are needed to maximize the engagement of mobile individuals in HIV care? Recognising the need for approaches that are responsive to diversity, that risk and opportunities for care may be affected by gender, age, sexual identity and ethnicity, is essential if hard to reach populations are to be reached by UTT. What possibilities exist for reconceptualising care delivery to address these challenges?

Mobile populations may be among those who stand to benefit the most from new models of differentiated care or differentiated service delivery, which aim to simplify and adapt HIV services across the cascade to better meet the needs of PLHIV and reduce burdens on health systems 63. These models include patient‐led community adherence groups, healthcare worker‐managed groups known as adherence clubs, fast‐track or multi‐month scripting, mobile outreach, and community drug distribution points. To the extent that these models can be informed by an understanding of the needs of mobile women and men living with HIV, they hold promise for engaging and retaining these populations who struggle to fit their needs to the requirements of clinic‐based HIV care systems.

In addition, mobile populations stand to benefit from improved therapeutic technologies such as long‐acting ART as well as longer‐acting formulations of biomedical prevention technologies such as Pre‐Exposure Prophylaxis (PrEP), and expansion of the delivery of these technologies beyond clinic settings into communities and key migration destinations and transit hubs. Structural and behavioural interventions to facilitate demand are needed to complement these ‘supply side’ interventions 2. Without such innovations, migrants will continue to be left behind in the quest to end the AIDS epidemic. We hope that this collection stimulates focus and commitment towards meeting this critical public health challenge.

## Competing interests

The authors have no competing interests to declare.

## Authors' contributions

CC, SC, and JS conceptualized the main messages. CC drafted the manuscript; SC and JS provided critical reviews. All authors read and approved of the final manuscript.
